# Bovine Milk Lactoferrin Selectively Kills Highly Metastatic Prostate Cancer PC-3 and Osteosarcoma MG-63 Cells *In Vitro*

**DOI:** 10.3389/fonc.2018.00200

**Published:** 2018-06-04

**Authors:** Joana P. Guedes, Cátia S. Pereira, Lígia R. Rodrigues, Manuela Côrte-Real

**Affiliations:** ^1^Center of Molecular and Environmental Biology (CBMA), Department of Biology, University of Minho, Braga, Portugal; ^2^Center of Biological Engineering (CEB), Department of Biological Engineering, University of Minho, Braga, Portugal

**Keywords:** highly metastatic cancer cells, V-ATPase, bovine lactoferrin, cancer therapy, intracellular pH, lysosomal dysfunction

## Abstract

Prostate cancer and osteosarcoma are the second most common type of cancer affecting men and the fifth most common malignancy among adolescents, respectively. The use of non-toxic natural or natural-derived products has been one of the current strategies for cancer therapy, owing to the reduced risks of induced-chemoresistance development and the absence of secondary effects. In this perspective, lactoferrin (Lf), a natural protein derived from milk, emerges as a promising anticancer agent due to its well-recognized cytotoxicity and anti-metastatic activity. Here, we aimed to ascertain the potential activity of bovine Lf (bLf) against highly metastatic cancer cells. The bLf effect on prostate PC-3 and osteosarcoma MG-63 cell lines, both displaying plasmalemmal V-ATPase, was studied and compared with the breast cancer MDA-MB-231 and the non-tumorigenic BJ-5ta cell lines. Cell proliferation, cell death, intracellular pH, lysosomal acidification, and extracellular acidification rate were evaluated. Results show that bLf inhibits proliferation, induces apoptosis, intracellular acidification, and perturbs lysosomal acidification only in highly metastatic cancer cell lines. By contrast, BJ-5ta cells are insensitive to bLf. Overall, our results establish a common mechanism of action of bLf against highly metastatic cancer cells exhibiting plasmalemmal V-ATPase. This study opens promising perspectives for further research on the anticancer role of Lf, which ultimately will contribute to its safer and more rational application in the human therapy of these life-threatening cancers.

## Introduction

Cancer is currently one the most lethal diseases worldwide, and metastases are the main cause of cancer-associated mortality. The urge to develop more targeted and efficient cancer therapies is therefore a current challenge (1). A recent study showed that the prostate cancer is one of the most frequently diagnosed cancers, being the second most common in men with incidence rates of 1.1 million (1). Also, despite its rarity, osteosarcoma is the most common primary bone malignancy in children and adolescents, and the fifth most common malignancy among adolescents and young aged (2, 3). Currently, the development of cancer therapy strategies based on the exploitation of different anticancer drugs, especially non-toxic natural or natural-derived products, has been subject of particular interest (4, 5). Among the different natural compounds with anticancer activities, some milk compounds and/or milk-derived bioactive peptides have been identified as potential agents for cancer prevention (6, 7). In particular, lactoferrin (Lf) is a natural iron-binding glycoprotein that was first identified in bovine milk. It is synthesized by mucosal epithelial cells and neutrophils during inflammatory processes and is present in many tissues and body fluids of mammals. Lf exhibits multiple biological effects, including anticancer and anti-metastatic activities against a wide range of human cancers (8–10). Lf from bovine milk [bovine lactoferrin (bLf)], which exhibits the same biological properties as the human Lf, is not only cheaply produced compared with other sources but is also commercially available and well tolerated after ingestion (11). Altogether, these properties confer to bLf the requirements of an ideal nutraceutical. In fact, the European Food Safety Authority has approved bLf as a safe ingredient for various applications (12). Interestingly, *in vitro* and *in vivo* studies, as well as clinical trials have been conducted to evaluate the effectiveness, safety, and tolerability of Lf in the treatment of metastatic cancers (13, 14). For instance, orally administered recombinant human Lf was well tolerated and displayed anticancer activity against solid tumors like non-small cell lung cancer and renal cell carcinoma, without secondary effects (13, 14).

Recent research has provided mechanistic insights on the anticancer activity of Lf based on its ability to interfere with cell cycle progression and to induce apoptosis (15, 16), as well as on its anti-metastatic (9, 17), anti-angiogenic (18), and immunostimulatory potential (19), and its iron sequestration capacity (20). Despite this knowledge, the molecular targets of Lf underlying its selective activity against cancer cells were until recently unknown.

However, we identified V-ATPase as a bLf target (21). V-ATPase is an ATP-driven proton pump that is normally present in the intracellular compartments (22) but, in highly metastatic cancer cells, it is also present at the plasma membrane and is responsible for the generation of an acidic tumor microenvironment, playing pivotal roles in tumor invasion and metastasis (23–25). In fact, previous studies showed that highly metastatic breast cancer cells express higher levels of V-ATPase, mainly localized at the plasma membrane, than poorly metastatic cancer cells, which display a predominant intracellular localization (23).

In our study, we assessed the sensitivity of breast cell lines with different metastatic potentials to bLf and showed that bLf exhibits preferential cytotoxicity against the highly metastatic cancer cell lines Hs 578T and MDA-MB-231, which display V-ATPase at the plasma membrane (21). These results supported the notion also reported by others (26) that this proton pump is an attractive target in the therapy of metastatic cancers and a promising candidate for anticancer drugs such as bLf.

Herein, we investigated the potential of bLf in the treatment of prostate cancer and osteosarcoma. To this end, we assessed its effect on cell proliferation and cell death in prostate PC-3 and osteosarcoma MG-63 highly metastatic cell lines, both reported to display V-ATPase at the plasma membrane (23–25), and compared it with the breast cancer MDA-MB-231 and the non-tumorigenic fibroblast BJ-5ta cell lines. Besides the effect of bLf on the intracellular pH (pHi), lysosomal acidification and extracellular acidification rate (ECAR), we also evaluated a possible relation between cell sensitivity and the V-ATPase protein levels in the four cell lines.

## Materials and Methods

### Chemical and Solutions

Bovine lactoferrin was obtained from DMV (Veghel, The Netherlands). The protein was dissolved in phosphate buffered saline (PBS) (1.37 M NaCl, 2.7 mM KCl, 10 mM Na_2_HPO_4_, 1.8 mM KH_2_PO_4_, pH 7.4) to achieve the different concentrations used throughout this study. According to the manufacturer, the protein purity is about 80% with 3.5% moisture and 21% iron-saturation. Concanamycin A (ConcA), paraformaldehyde, cisplatin, etoposide, and β-actin antibody were purchased from Sigma-Aldrich. Lysosensor Green DND-189 and BCECF-AM [2′, 7′-bis-(2-carboxyethyl)-5-(and-6)-carboxyfluorescein, acetoxymethyl ester] were obtained from Molecular Probes. Carboxyfluorescein diacetate succinimidyl ester (CFSE) probe and FITC Annexin V apoptosis detection kit were acquired from BD Bioscience. Secondary antibody anti-mouse IgG was obtained from Jackson ImmunoResearch. V-ATPase C1 antibody was purchased from Santa Cruz Biotechnology. Vectashield mounting medium was acquired from Biosystems.

### Cell Lines and Culture Conditions

Human prostate cancer cell line PC-3 (CRL-1435; ATCC), human osteosarcoma cell line MG-63 (CRL-1427; ATCC), and human breast cancer cell line MDA-MB-231 (HTB-26; ATCC) were grown in Dulbecco’s Modified Eagle’s Medium (DMEM), supplemented with 10% fetal bovine serum (FBS), both purchased from Biochrom—Merck Millipore, and 1% zell shield (Minerva Biolabs). Human fibroblast cell line BJ-5ta (CRL-4001; ATCC) was grown in a 4:1 mixture of DMEM and Medium 199, purchased from Biochrom—Merck Millipore, supplemented with 0.01 mg/ml hygromycin B (Sigma-Aldrich) and 10% FBS. Cells were maintained in culture in a 37°C incubator with a humidified atmosphere containing 5% CO_2_. For each experiment, cells were seeded in 6-well plates at appropriate concentrations: 1 × 10^5^ at 24 h and 7.5 × 10^4^ cell/ml at 48 h for the PC-3 cell line; 9 × 10^4^ at 24 h and 5 × 10^4^ cell/ml at 48 h for MG-63 and MDA-MB-231 cell lines; and 1.5 × 10^5^ at 24 h, 1 × 10^5^ at 48 h, and 9 × 10^4^ cell/ml at 72 h for the BJ-5ta cell line. For ECAR experiments, cells were seeded in XF 24-well plates at a concentration of 1 × 10^4^ cells/well. All the compounds under study were added to the wells only when cells reached at least 70% confluence.

### Assessment of Cell Proliferation With CFSE

Carboxyfluorescein diacetate succinimidyl ester labeling was performed before cell seeding. Briefly, cells were collected from the culture flask, washed with 1× PBS and incubated with the CFSE dye (final concentration: 20 µM) for 15 min in a 37°C water bath. Afterward, cells were rinsed with 1× PBS, the correct amount of complete culture media to obtain the same cell concentration was added, and cells were plated in 6-well plates. After adhering for 24 h protected from light, cells were treated with medium (negative control), 50 µM cisplatin (MG-63 and MDA-MB-231 cell lines) or 60 µM etoposide (PC-3 and BJ-5ta cell lines) as positive controls, as well as 175 µM bLf. Cells were harvested and the carboxyfluorescein (CF) median fluorescence intensity was analyzed by flow cytometry using the FL1 channel 0, 24, 48, and 72 h after treatment. At the moment of seeding, a sample from the labeled cell suspension was also collected and analyzed to ensure correct cell staining. All data (median values) were normalized to the time point 0 h, which corresponds to the maximum fluorescence intensity.

### FITC Annexin V/Propidium Iodide (AV/PI) Apoptosis Assay

Cells were seeded in 6-well plates, two wells per condition—negative control (without treatment), positive control (60 µM etoposide for PC-3 and BJ-5ta cell lines or 50 µM cisplatin for the MG-63 cell line), 175 µM bLf and 10 nM ConcA (used as positive control for V-ATPase inhibition) and incubated for 48 and/or 72 h. Apoptosis was detected using the “FITC Annexin V apoptosis detection kit” according to the manufacturer’s instructions (BD Biosciences). After treatment, 2 × 10^5^ cells per condition were harvested, rinsed with 1× PBS, resuspended in 1× Binding Buffer (BD Pharmigen™) and incubated with 1 µl of AV-FITC and 1 µl of PI for 15 min in the dark. Acquisition was performed in a flow cytometer using the FL1 and FL4 channels, respectively.

### pHi Measurement

Measurements of pHi were performed with the pH-sensitive probe BCECF-AM. Cells were seeded in 6-well plates and treated with medium alone, 175 µM bLf or 10 nM ConcA, for 24 and/or 48 h. After this time, cells were tripsinized and washed with Hank’s balanced salt solution (HBSS) (10× concentrated solution: 1,379 mM NaCl; 53.3 mM KCl; 3.4 mM Na_2_HPO_4_-7H_2_O; 55.6 mM d-glucose and 4.4 mM KH_2_PO_4_). The pellets were resuspended in 1× HBSS and incubated with 50 µM BCECF-AM from a stock solution of 161.3 µM for 30 min at 37°C protected from light. Samples were analyzed in a flow cytometer. The percentage of cells exhibiting intracellular acidification was estimated from the percentage of cells displaying a FL1/FL4 ratio lower than control cells. This ratio is independent of the probe concentration and exclusively dependent on the pHi.

### ECAR Measurement

Basal ECARs of MDA-MB-231, PC-3, MG-63, and BJ-5ta cell lines were determined using a Seahorse Extracellular Flux (XF24) Analyzer (Seahorse Bioscience). Cells were seeded into XF24 cell culture microplates at a cellular density of 1 × 10^4^ cells/well in their normal growth media, left to adhere overnight in a humidified 37°C incubator with 5% CO_2_ and then treated with 175 µM bLf for 24 h. In the wells corresponding to the negative control, the medium was changed and no treatment was added. Prior to the basal ECAR measurement, the growth medium was exchanged to a basal assay medium (DMEM 5030—Sigma-Aldrich) supplemented with 4 mM glutamine and rigorously adjusted to pH 7.4 ± 0.1. Then, the plates were incubated for 1 h in a 37°C/non-CO_2_ incubator to deplete all the glycolytic reserves. After ECAR measurements, the amount of protein present in each well was estimated using the sulforhodamine B (SRB) assay since each cell line has a different proliferation rate during the incubation period. ECAR values were normalized to the SRB absorbance of each well using the Wave 2.2.0 software, and plotted as the mean ± SD, each point representing the average of different wells.

### Assessment of Cell Proliferation by SRB Assay

Cells were seeded in 6-well plates and incubated with 175 µM bLf for 24 h. Afterward, cells were fixed for 90 min at −20°C in ice-cold 1% acetic acid in methanol, and then incubated with 0.5% (w/v) SRB in 1% acetic acid for 90 min at 37°C. After washing with 1% acetic acid and drying, protein-bound SRB was dissolved in 10 mM Tris pH 10.5 for 10 min at room temperature (RT). A sample from each condition was transferred to a 96-well plate and absorbance was read at 540 nm in a microplate reader (SpectraMax 340PC, Molecular Devices).

### Protein Quantification

The soluble protein concentration was determined using the Bio-Rad DC protein assay (Bio-Rad Laboratories), by a modified Lowry method. The protein concentration of each sample was determined using a calibration curve obtained with solutions of BSA with increasing concentrations—from 5 to 0.25 mg/ml.

### Western Blot

For western blot, 35 µg of protein were separated by 12.5% sodium dodecyl sulfate-polyacrylamide gel electrophoresis. Afterward, proteins were transferred onto polyvinylidene difluoride membranes. Next, to avoid non-specific interactions, membranes were blocked in 5% non-fat milk in PBS-Tween 0.1% solution (1× PBST) with agitation at RT for 1–2 h. Membranes were then incubated overnight at 4°C with the primary antibodies, namely monoclonal anti-β-actin and anti-V-ATPase. Subsequently, membranes were incubated with secondary antibody goat anti-mouse IgG (1:2,000). Chemiluminescence detection was performed using the ECL detection system (Millipore-Merck) in a ChemiDoc^TM^ XRS system (Bio-Rad).

### Measurement of Lysosomal Acidification

Measurements of lysosomal acidification were performed with the Lysosensor Green DND-189 probe. Cells were grown in glass coverslips in 6-well plates and, after 24 h of adherence, were incubated with 175 µM bLf or 10 nM ConcA for 48 h. Then, the medium was removed and pre-warmed 1 µM probe-containing medium was added during 30 min, in a 37°C incubator with a humidified atmosphere containing 5% CO_2_. Next, the medium was replaced with fresh medium and the coverslips were mounted upside down in Vectashield. Samples were visualized in a Leica DM 5000B (Leica Microsystems) fluorescence microscope with specific filter settings for Lysosensor Green. Cells from the same wells that were attached to the well surface and not the coverslip were tripsinized and analyzed by flow cytometry using the FL1 channel. The % of cells exhibiting lysosomal acidification was estimated from the percentage of cells displaying a decreased fluorescence intensity in comparison to control cells.

### Flow Cytometry Analysis

Flow cytometry analysis was performed in an Epic^®^ XL^TM^ (BeckmanCoulter) flow cytometer equipped with an argon-iron laser with emission of a 488 nm beam at 15 mW. Red fluorescence was collected through a 560 nm short-pass dichroic, a 640 nm long-pass, and another 670 nm long-pass filter. Green fluorescence was collected through a 488 nm blocking filter, 525 nm band-pass filter, and a 550 nm long-pass dichroic. For each experiment, 20,000 events were evaluated for each sample and data were analyzed using the FlowJo 7.6 software.

### Statistical Analysis

Experimental values are expressed as means or medians ± SD of at least three independent experiments. Statistical analysis was performed using one-way ANOVA followed by the Bonferroni post-test using the GraphPad Prism version 6.0.

## Results

### The Highly Metastatic Prostate Cancer PC-3 and the Osteosarcoma MG-63 Cell Lines Are Sensitive to bLf

**Figure 1:**
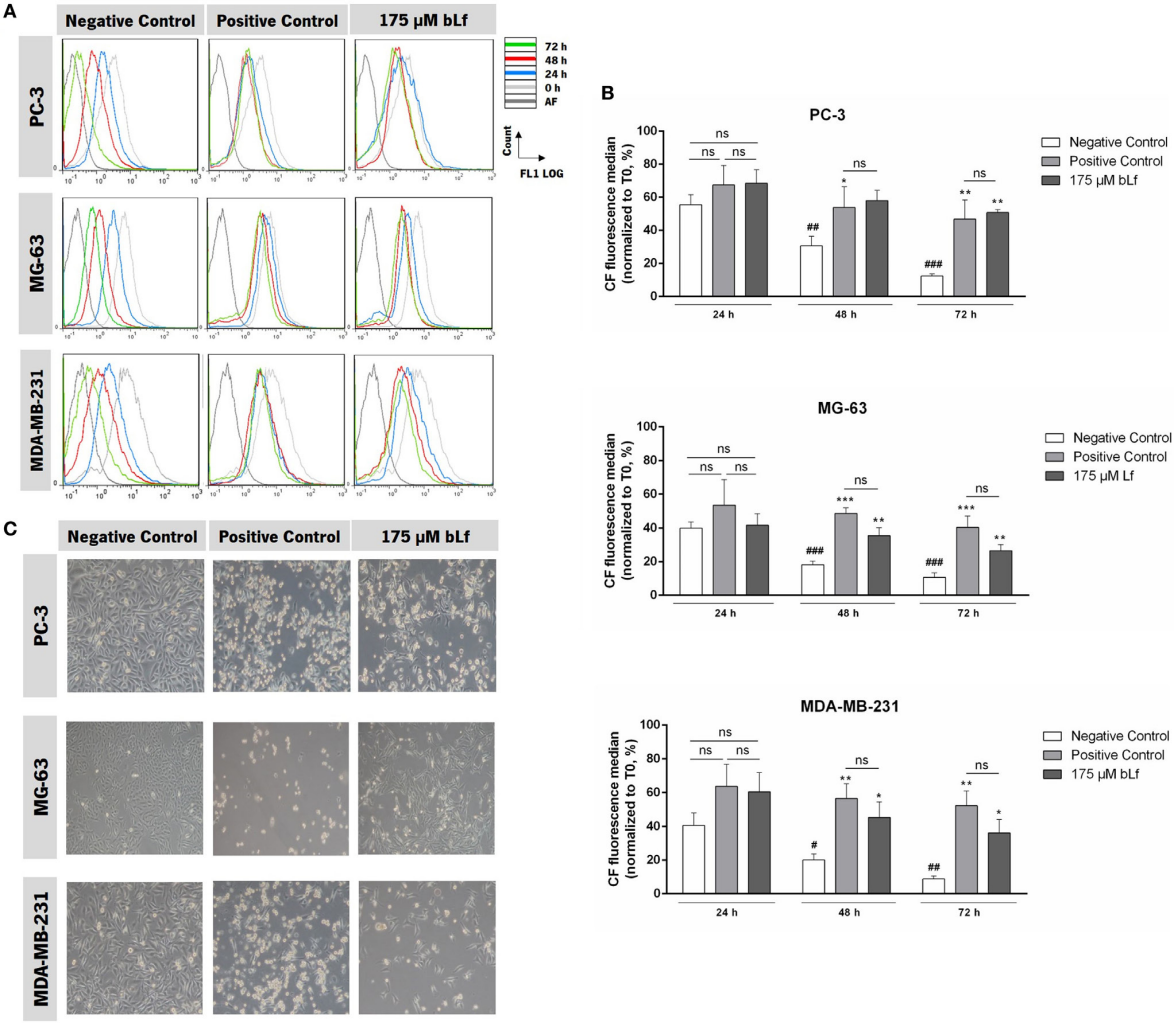
Bovine lactoferrin (bLf) inhibits proliferation of highly metastatic cancer cell lines. **(A)** Representative histograms of cell proliferation analysis with carboxyfluorescein diacetate succinimidyl ester probe of the different conditions after 0, 24, 48, and 72 h exposure to bLf in the three indicated cell lines. Unexposed cells were used as a negative control and 50 µM cisplatin (for MG-63 and MDA-MB-231 cell lines) or 60 µM etoposide (for PC-3 cells) were used as positive controls. **(B)** Quantification of the carboxyfluorescein median fluorescence intensity values normalized to T0 after 24, 48, and 72 h of exposure. Values represent median ± SD of three independent experiments. *P < 0.05, **P < 0.01, and ***P < 0,001 compared with negative control of each time point and ^#^*P* < 0.05; ^##^*P* < 0.01, and ^###^*P* < 0,001 compared with 24 h. **(C)** Representative images of cell cultures after 48 h of treatment with bLf, cisplatin or etoposide in PC-3, MG-63, and MDA-MB-231 cell lines (10× phase contrast).

To test whether the highly metastatic prostate cancer PC-3 and the osteosarcoma MG-63 cell lines were sensitive to bLf, we optimized a CFSE staining protocol and used the bLf-sensitive highly metastatic breast cancer cell line MDA-MB-231 for comparison. In each cell division, the fluorescence of CFSE-stained cells is reduced to half of the initial fluorescence (27). Thus, when cell proliferation is inhibited, the expected decrease of intracellular fluorescence is not observed. We found that cell fluorescence 24 h after incubation with bLf, etoposide, or cisplatin—the latter used as positive controls for PC-3 and MG-63, respectively—was not significantly different from that of untreated cells (negative control) (Figures 1A,B). However, after 48 and 72 h of incubation with bLf, as well as with the positive controls, the cell fluorescence decreased much slower than in the control cells (Figures 1A,B), indicating that cell proliferation was inhibited. The inhibition of cell proliferation (%) by bLf or by etoposide/cisplatin for each cell line was estimated and no significant differences between the three cancer cell lines were found (Table S1 in Supplementary Material). These results indicate that PC-3 and MG-63 cells exhibit a sensitivity to bLf similar to that observed for the well-known apoptotic inducers, cisplatin or etoposide. A similar result was obtained for MDA-MB-231 cells that, in our previous study, showed equally a high sensitivity to bLf (21). In accordance with the observed inhibition of cell proliferation measured 48 h after exposure to bLf, a decrease in the cell number and changes in cell morphology were observed by phase contrast microscopy (Figure 1C).

### bLf Induces Apoptosis of PC-3 and MG-63 Cancer Cell Lines

**Figure 2:**
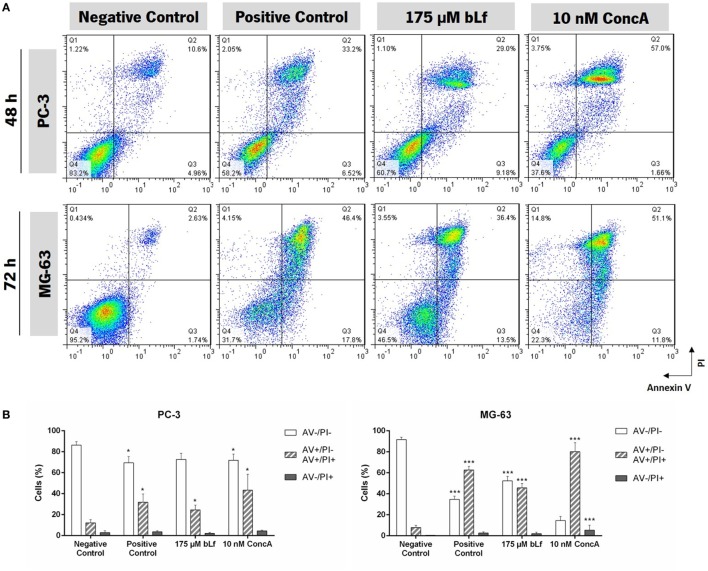
Bovine lactoferrin (bLf) induces cell death of the highly metastatic cancer cell lines PC-3 and MG-63. **(A)** Exposure of phosphatidylserine induced by bLf or concanamycin A (ConcA) monitored by AV-FITC/PI staining. Representative histograms of cells untreated (negative control) or treated during 48 h (for PC-3) and 72 h (for MG-63) with 175 µM bLf or 10 nM ConcA, or with 50 µM cisplatin or 60 µM etoposide (used as positive controls, for MG-63 and PC-3 cells, respectively). **(B)** Quantitative analysis of the AV/PI assays in panel **(A)**. Values represent mean ± SD of three independent experiments, *P < 0.05 and ***P < 0.001 when compared with negative control cells.

The observed decrease in proliferation of bLf-treated cells can be due to inhibition of cell division *per se* or to induction of cell death, specifically of apoptosis. To address this hypothesis, we evaluated whether bLf was able to induce exposure of phosphatidylserine (PS) to the outer leaflet of the plasma membrane while preserving the plasma membrane integrity (28) using the FITC-AV/PI staining assay. Figure 2A illustrates representative biparametric histograms of the cells treated with cisplatin, etoposide, bLf, or the V-ATPase inhibitor ConcA. Results showed that incubation with bLf, as well as with etoposide or ConcA, led to exposure of PS in PC-3 cells. Indeed, the percentage of early (AV+/PI−) and late apoptotic cells (AV+/PI+) for this cell line increased after 48 h of incubation with these compounds in comparison with untreated cells. On the other hand, no early or late apoptotic MG-63 cells could be detected after 48 h of treatment. However, we found that bLf, like cisplatin, induced exposure of PS 72 h after exposure, indicating induction of apoptosis (Figures 2A,B). Moreover, the percentage of AV−/PI+ cells for the two cell lines was very low under all treatment conditions.

### bLf Does Not Affect Cell Proliferation Nor Induces Cell Death of the Non-Tumorigenic Cell Line BJ-5ta

**Figure 3:**
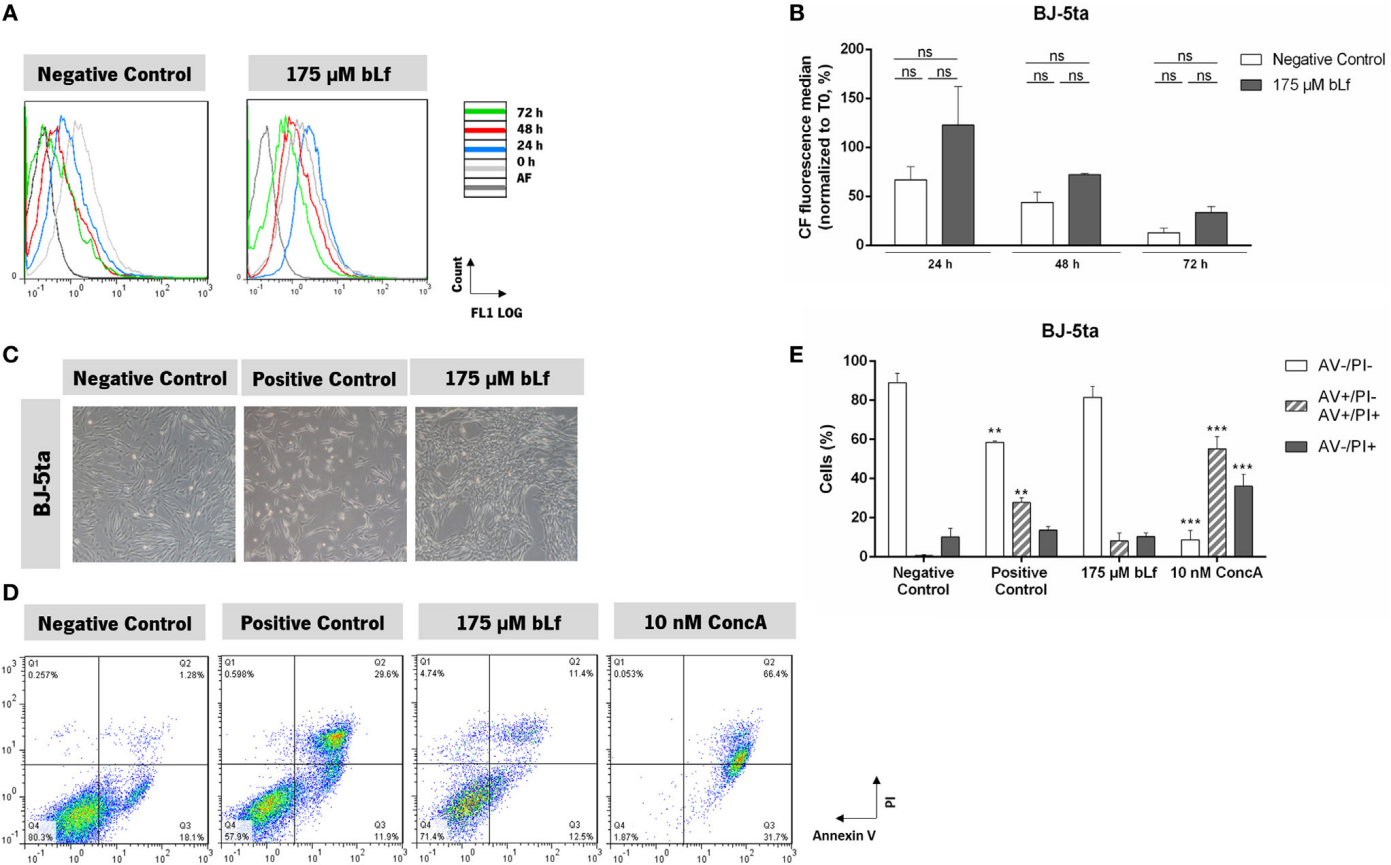
Bovine lactoferrin (bLf) does not affect the cell proliferation nor induces cell death on the non-tumorigenic cell line BJ-5ta. **(A)** Representative histograms of cell proliferation analysis with carboxyfluorescein diacetate succinimidyl ester probe after 0, 24, 48, and 72 h exposure to bLf. Unexposed cells were used as a negative control. **(B)** Quantification of the carboxyfluorescein median fluorescence values normalized to T0 after 24, 48, and 72 h of exposure. Values represent median ± SD of three independent experiments. ns, non-significant. **(C)** Representative images of BJ-5ta cell cultures after 48 h of treatment with bLf and etoposide (10× phase contrast). **(D)** Exposure of phosphatidylserine induced by bLf or concanamycin A (ConcA) was monitored by AV-FITC/PI staining. Representative histograms of untreated cells (negative control) or treated during 72 h with 175 µM bLf, 10 nM ConcA, or with 60 µM etoposide (used as positive control). **(E)** Quantitative analysis of the AV/PI assays in panel **(D)**. Values represent mean ± SD of three independent experiments, **P < 0.01 and ***P < 0.001 when compared with negative control cells.

To assess whether bLf was selective against highly metastatic cancer cell lines, we next analyzed its effect on the inhibition of cell proliferation and induction of cell death in the non-tumorigenic cell line BJ-5ta. We observed that, after 72 h, bLf-treated cells exhibited an intracellular CFSE fluorescence decrease similar to that of untreated cells, indicating that cell proliferation was not affected and that the BJ-5ta cell line is not sensitive to bLf (Figures 3A–C). This was further confirmed by assessing apoptosis induction with the AV/PI assay. Figure 3D illustrates representative biparametric histograms of cells treated with bLf, etoposide, or ConcA for 72 h. Exposure of PS (AV+/PI− and AV+/PI+ cells) was only significantly different from the control cells after incubation with etoposide or ConcA. By contrast, these percentages were not significantly different between untreated and bLf-treated cells, suggesting that, at this time point, bLf does not induce apoptosis (Figure 3E).

### bLf Induces Intracellular Acidification and Inhibits the ECAR in PC-3 and MG-63 Cancer Cell Lines

**Figure 4:**
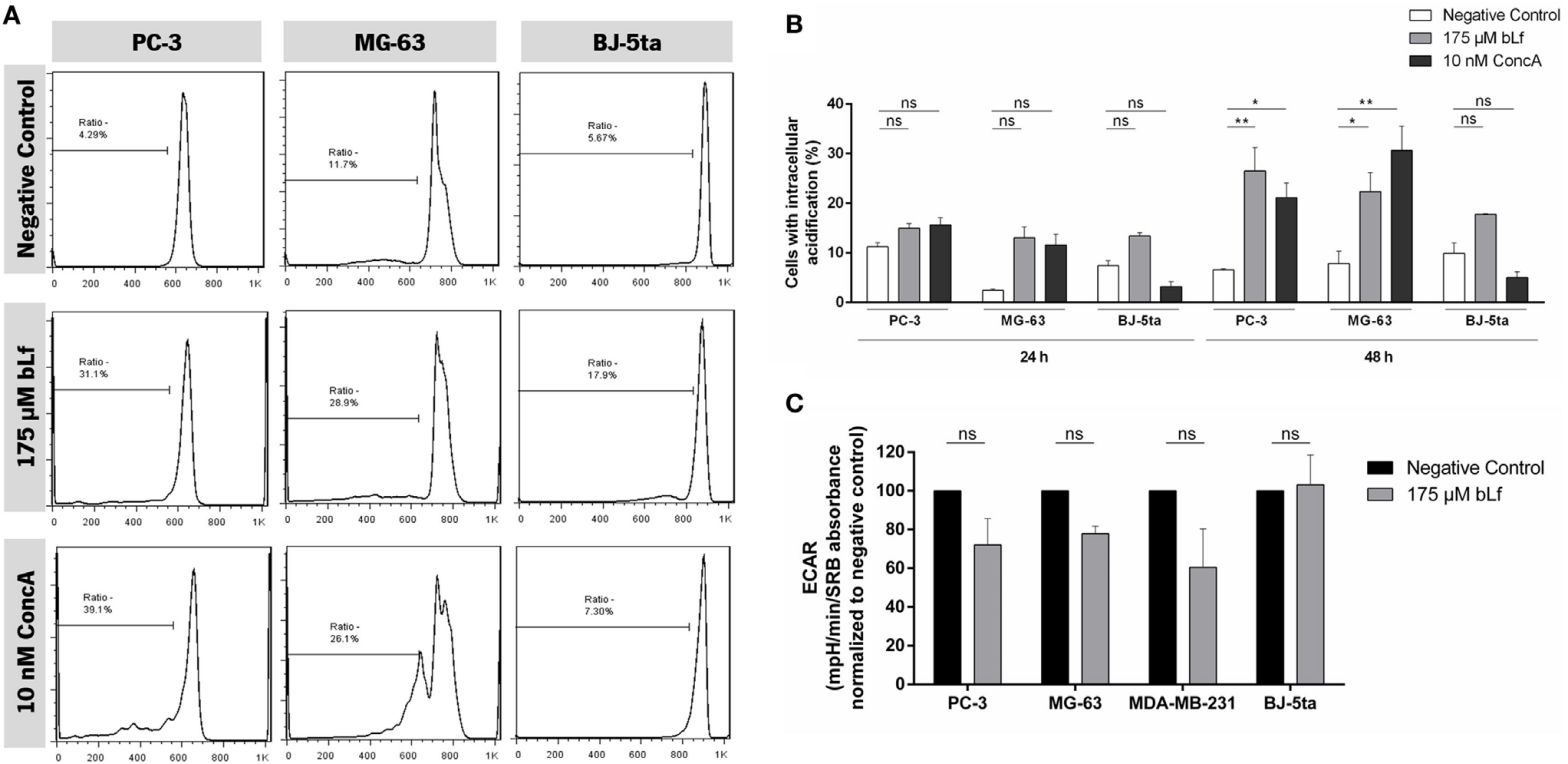
Effect of bovine lactoferrin (bLf) on the intracellular acidification and basal extracellular acidification rates (ECARs) in the highly metastatic cancer cell lines PC-3 and MG-63 and in the non-tumorigenic cell line BJ-5ta. **(A)** Representative histograms of the percentage of BCECF-AM-loaded cells displaying intracellular acidification assessed through the decrease of FL1/FL4 median fluorescence intensity ratio in comparison to negative control cells at 48 h. **(B)** Quantification of the percentage of cells displaying intracellular acidification after treatment with 175 µM bLf or 10 nM concanamycin A (ConcA), for 24 and 48 h (identified under the cursor on ratio). Values represent the mean ± SD of three independent experiments; ns, non-significant, *P < 0.05 and **P < 0.01 compared with the negative control of each cell line. **(C)** Basal ECARs of cells after 24 h incubation in the absence (negative control) or presence of 175 µM bLf. The bLf sensitive highly metastatic breast cancer cell line was used for comparison. ECAR values are normalized to the negative control cells of each cell line. Results are expressed in mpH per minute per sulforhodamine B (SRB) absorbance at 540 nm. Values represent the mean ± SD of three independent experiments.

We next evaluated whether the observed cytotoxic effects of bLf could be associated with an intracellular acidification and a decrease in the capacity of PC-3 and MG-63 cancer cells to acidify the extracellular medium due to the inhibition of V-ATPase by bLf. To this end, pHi measurements were performed using the pH-sensitive probe BCECF-AM. This probe rapidly diffuses into cells and is cleaved by intracellular esterases to its unsterified form, which emits fluorescence according to the pHi (29). The results are expressed as the percentage of cells that exhibit a decrease in the ratio of FL1/FL4 (linear cursor), reflecting an intracellular acidification in comparison with untreated cells (negative control). Figure 4A shows representative histograms of cells incubated in the absence or presence of 175 µM of bLf or 10 nM ConcA for 48 h. We found an increase in the percentage of PC-3 and MG-63 cells exhibiting intracellular acidification after 48 h of treatment with bLf or ConcA, in comparison with untreated cells (Figures 4A,B). By contrast, the bLf or ConcA-treated BJ-5ta cells did not exhibit alterations of pHi, indicating a similar behavior to untreated cells.

Basal ECAR measurements were performed using an XF Extracellular Flux Analyzer, which allows measuring the non-glycolytic acidification often named basal ECAR. Before recording the ECAR, 24 h-treated cells were transferred to a glucose-free medium and incubated for 1 h in a non-CO_2_ incubator to exhaust their glycolytic reserves. During this period, the production and efflux of lactic acid occurs, which ensures that the subsequent measurement of proton extrusion only reflects the extracellular acidification mediated by the plasmalemmal V-ATPase. Though not statistically significant, results showed that a 24-h treatment with 175 µM bLf inhibited the basal ECAR of the prostate PC-3 and osteosarcoma MG-63 cancer cells as well of the breast cancer cells MDA-MB-231, while had no effect on the non-tumorigenic cells (Figure 4C).

### The High Sensitivity of PC-3 and MG-63 Cells to bLf Is Associated With High Levels of V-ATPase Expression

**Figure 5:**
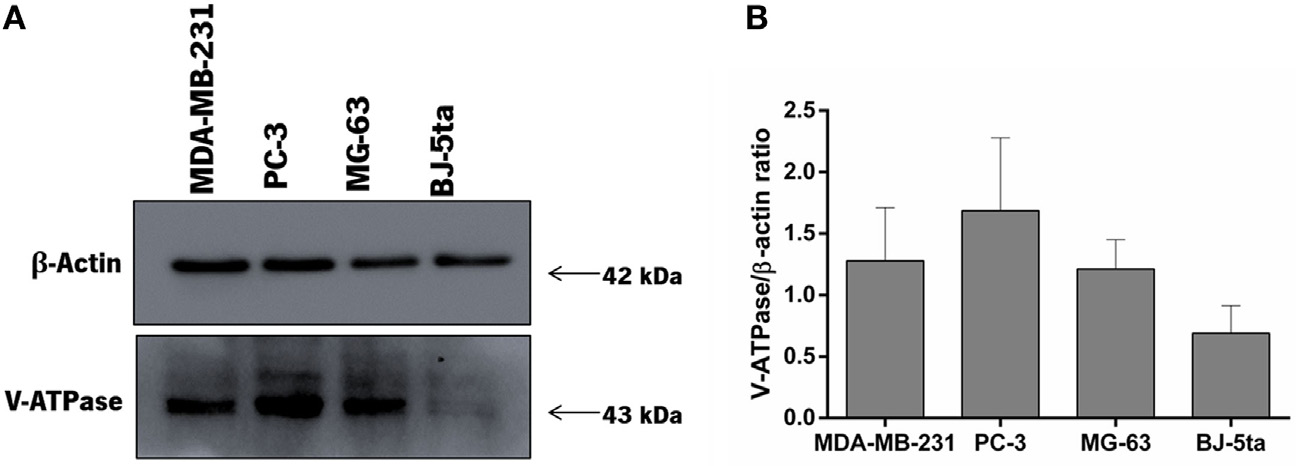
V-ATPase expression levels in highly metastatic prostate PC-3 and osteosarcoma MG-63 cancer cell lines in comparison with the highly metastatic breast cancer cell line MDA-MB-231 and the non-tumorigenic cell line BJ-5ta. **(A)** Representative western blot images of V-ATPase expression in each cell line using 35 µg of protein extracts in each lane. β-actin was used as a loading control. **(B)** Quantification of the levels of V-ATPase expression in the four cell lines normalized to the levels of β-actin. The values represent three independent assays.

In order to determine a possible relationship between the different sensitivity of highly metastatic cancer cells and non-tumorigenic cells to bLf and differences in V-ATPase expression, the levels of this proton pump were evaluated by western blot (Figure 5). Results showed that PC-3 and MG-63 cells display higher levels of V-ATPase in comparison with the non-tumorigenic cell line BJ-5ta. We also observed that the total amount of the V-ATPase is higher in the three highly metastatic cancer cells than in the non-tumorigenic cell line BJ-5ta, which is in agreement with previous results showing that in these cells V-ATPase is highly abundant in the plasma membrane (23–25).

### bLf Inhibits Lysosomal Acidification in PC-3, MG-63, and MDA-MB-231 Cancer Cell Lines but Not in the Non-Tumorigenic Cell Line BJ-5ta

**Figure 6:**
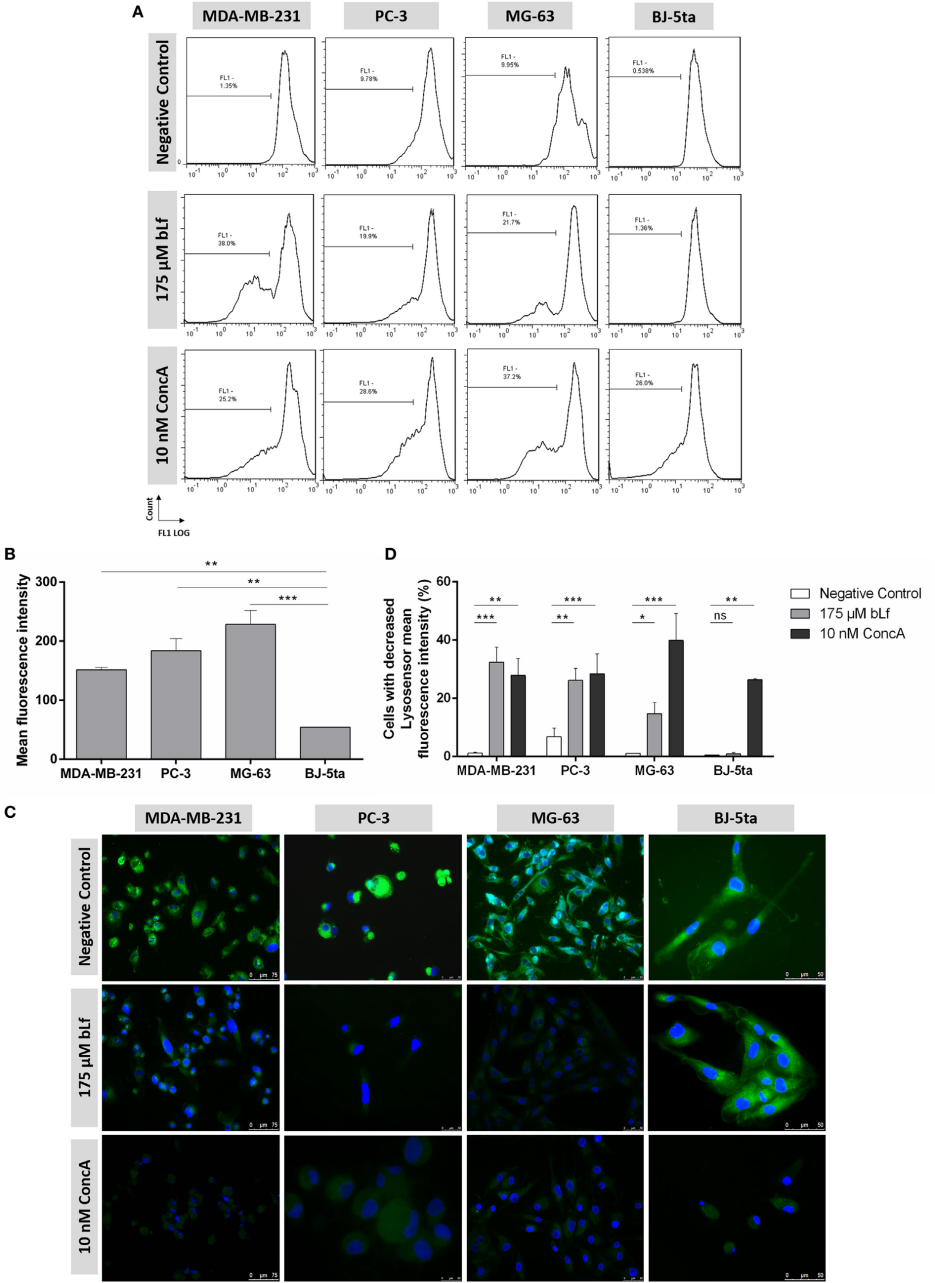
Bovine lactoferrin (bLf) inhibits lysosomal acidification in the highly metastatic cancer cell lines but not in the non-tumorigenic cell line. **(A)** Representative histograms of the % of Lysosensor-loaded MDA-MB-231, PC-3, MG-63, and BJ-5ta cells displaying lower FL1 mean fluorescence intensity (FL1-cursor) after 48 h treatment with 175 µM bLf or 10 nM ConcA. **(B)** Mean fluorescence intensity values of the four cell lines before treatment with bLf. Values represent the mean ± SD of three independent experiments; **P < 0.01 and ***P < 0.001 compared with BJ-5ta cells. **(C)** Representative fluorescence microscopy images of the cells stained with Lysosensor Green DND-189 upon 48 h of treatment with 175 µM bLf or 10 nM ConcA. The probe stains acidic compartments mainly lysosomes. **(D)** Mean % values of cells treated under the same conditions displaying lower fluorescence intensity in comparison with untreated cells. Values represent the mean ± SD of three independent experiments; *P < 0.05, **P < 0.01, and ***P < 0.001 compared with the negative control of each cell line.

Different studies showed that compounds with a similar effect to the well-established V-ATPase inhibitors bafilomycin A1 (BafA1) and ConcA prevent lysosomal acidification in mammalian cells (30). We therefore evaluated whether exposure to bLf, as well as to ConcA, could inhibit the lysosomal V-ATPase in the cell lines under study. For this purpose, cells were stained with Lysosensor Green, a fluorescent dye that specifically stains acidic organelles such as lysosomes, and has a pH-dependent fluorescence intensity that decreases upon alkalinization of acidic compartments. Flow cytometric analyses of the untreated cells of the three highly metastatic cancer cell lines revealed a more acidic lysosomal compartment in comparison to the non-tumoral cells. Indeed, the median intensity fluorescence values of these cells were threefold to fourfold higher than BJ-5ta cells (Figures 6A,B). This strongly suggests that V-ATPase is more abundant (or more active) in the lysosomal membrane of highly metastatic cancer cells. We also observed by fluorescence microscopy that the three highly metastatic cancer cell lines exhibited a decrease in fluorescence intensity 48 h after treatment with bLf or ConcA, in contrast with untreated cells (negative control) (Figure 6C). On the other hand, bLf has no effect against BJ-5ta cell line, in contrast with ConcA that decreased the fluorescence intensity. These results were further confirmed by monitoring the percentage of Lysosensor-loaded cells displaying lower median fluorescence intensity values by flow cytometry (Figures 6A,D). Accordingly, bLf induced an increase in the percentage of cells with lower fluorescence intensity in the highly metastatic cancer cells but not in the non-tumorigenic cells while ConcA decreased the fluorescence in all cell lines (Figures 6C,D).

## Discussion

Prostate cancer and osteosarcoma are incident and relevant causes of human deaths due to different factors. The rising trends in prostate cancer incidence and mortality have been largely attributable to the widespread availability of prostate-specific antigen tests in the late 1980s and hence to the increased detection of latent disease (31). In the case of osteosarcoma, there is an increased risk of developing childhood cancer following radiotherapy or treatment with alkylating agents (2). Therefore, the finding and characterization of novel efficient drugs is a current challenge. Since bLf is a natural anticancer compound that has no secondary effects and reduced risk of chemoresistance, we addressed whether it may be exploited in the therapy of these cancers. For this purpose, we determined the activity of bLf against the highly metastatic prostate cancer (PC-3) and osteosarcoma (MG-63) cell lines. In our previous study, we tested different bLf concentrations—50, 125, and 175 µM and found that the effect was more evident with 175 µM bLf (21). In this sense, in this study, we only used this concentration. Herein, we show that PC-3 and MG-63 cells are highly sensitive to bLf, regarding both inhibition of cell proliferation, induction of apoptosis and intracellular acidification. Notably, these cell lines that have high sensitivity to bLf have V-ATPase localized at the plasma membrane (21, 23–25). We also show that bLf tends to decrease the basal ECAR, which is mainly maintained by V-ATPase in highly metastatic cancer cells (23). By contrast, none of these effects were detected in the non-tumorigenic BJ-5ta cells. Thus, exposure of V-ATPase at the plasma membrane and its inhibition in the prostate cancer and osteosarcoma cells seems to determine their sensitivity to bLf, as previously shown for the highly metastatic breast cancer cell lines (21).

To further support that V-ATPase is a molecular target of bLf in PC-3 and MG-63 cancer cells, we examined a possible relation between cell sensitivity and the levels of V-ATPase as compared with the bLf-sensitive breast cancer MDA-MB-231 and to the bLf-insensitive non-tumorigenic BJ-5ta cell lines. We show that the higher sensitivity of the three highly metastatic cell lines to bLf is associated with higher levels of V-ATPase in comparison with the non-tumorigenic cell line. Accordingly, other authors have also reported that cancer cells have increased levels of V-ATPase (32, 33). These results support the notion that bLf targets the V-ATPase at the plasma membrane, hindering its activity and decreasing the TME acidity, and in this way likely limit tumor progression and metastasis.

It was reported that PC-3 cells undergo apoptosis when exposed to other compounds like the chemotherapeutic agents salinomycin (34) and monensin (35), associated with exposure of PS. Also, other studies with the osteosarcoma MG-63 cell line showed apoptosis induction through exposure of PS by treatment with berberine (36) and ascorbic acid (37). Herein, we demonstrate that bLf-induced inhibition of cell proliferation in the highly metastatic cancer cell lines PC-3 and MG-63 is associated with an increase in AV+/PI− staining. Similarly, we recently showed that cell proliferation inhibition of the highly metastatic breast cancer cells Hs 578T and MDA-MB-231 by bLf was accompanied by induction of cell death associated with exposure of PS (21). Other authors found that the non-invasive breast cancer cell line MCF-7, sensitive to bLf, also displays this apoptotic marker in response to bLf treatment (8, 9). Furthermore, in contrast to ConcA or etoposide-treated cells, the non-tumorigenic cells BJ-5ta do not die even 72 h after exposure. This observation together with the lack of inhibition of cell proliferation is in good agreement with their resistance to bLf. Likewise, we also did not detect early and late apoptotic cells for the non-tumorigenic breast cell line MCF-10-2A (21).

**Figure 7:**
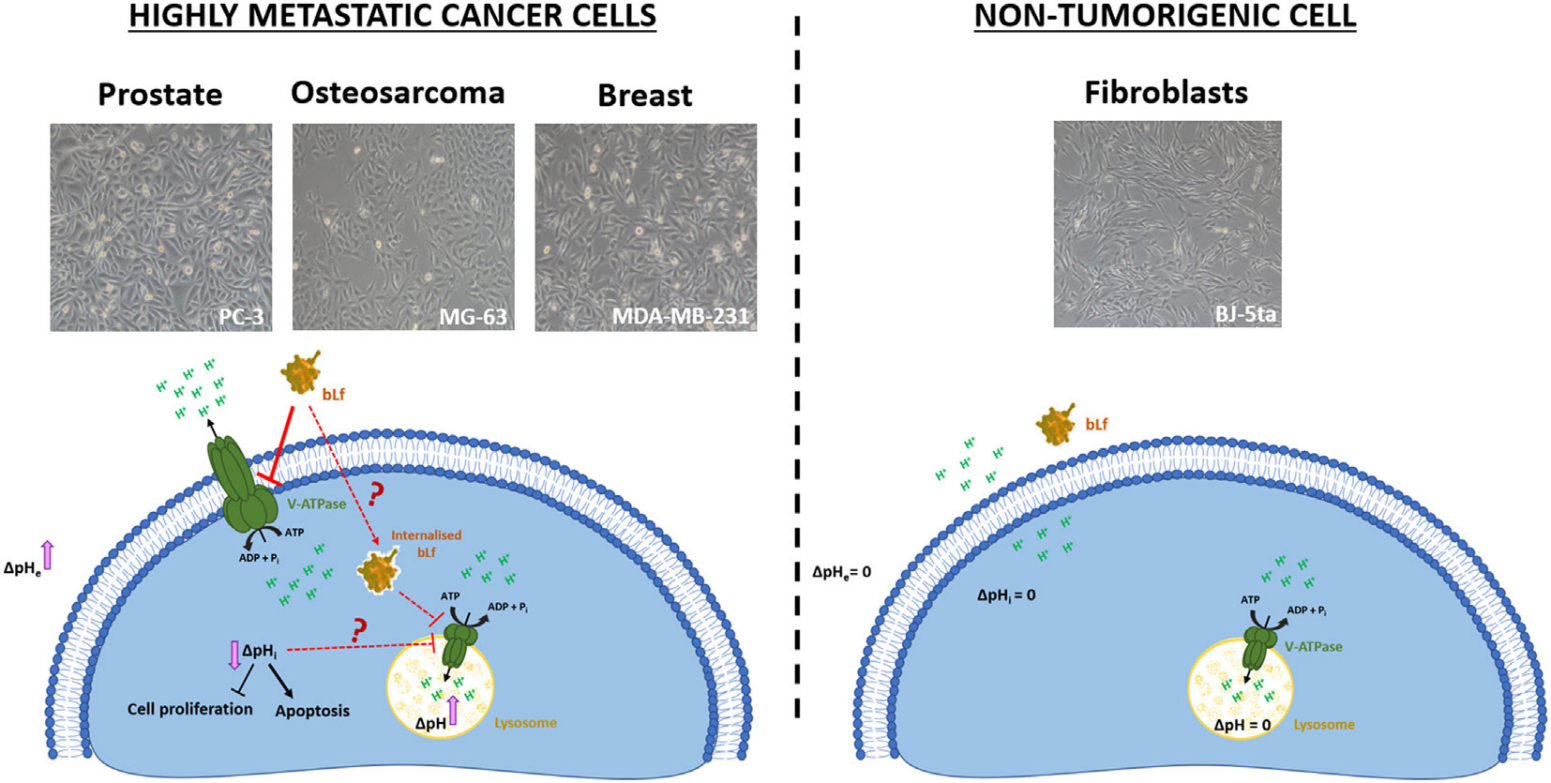
Working model for the molecular mechanism underlying the anticancer activity of bovine lactoferrin (bLf). The exposure of highly metastatic cancer cells to bLf leads to the inhibition of plasmalemmal V-ATPase proton pumping activity and subsequent extracellular alkalization, intracellular acidification, inhibition of cell proliferation, and induction of apoptosis. The observed lysosomal dysfunction is possibly due to lysosomal V-ATPase inhibition. By contrast, the non-tumorigenic cells, which do not have plasmalemmal V-ATPase and express lower levels of this protein, are resistant to bLf.

As referred above, BafA1 and ConcA interfere with V-ATPase proton pumping activity and affect lysosomal acidification in mammalian cells (30). Interestingly, a recent study reported the sensitivity of the human breast cancer cells MDA-MB-231 to the proton pump inhibitor lansoprazole (38). These authors showed that this compound induces apoptosis through its ability to suppress proton pumping activity and induce lysosomal alkalinization. We therefore evaluated whether exposure to bLf, as well as to ConcA, could inhibit the lysosomal V-ATPase in the cell lines under study. We show that, as described for lansoprazole in breast cancer cell lines, bLf induces lysosomal pH perturbations similarly to ConcA, in the highly metastatic cancer cell lines PC-3, MG-63, and MDA-MB-231. However, the BJ-5ta cells treated with bLf, but not with ConcA, exhibit a behavior identical to that of untreated cells. It is noteworthy that PC-3, MG-63, and MDA-MB-231 cells show lysosomes with lower pH than BJ-5ta cells. This is likely due to a higher V-ATPase content at the lysosomal membrane, which would explain the relation between lysosomal dysfunction and sensitivity to bLf. This perturbation in lysosomal pH is much likely due to the intracellular acidification mediated by inhibition of the plasmalemmal V-ATPase or possibly by internalized bLf. Indeed, though there is no data in the literature regarding internalization of bLf in the prostate PC-3 and osteosarcoma MG-63 cell lines, in the case of MDA-MB-231 cells the internalization of a small amount of bLf was reported (17). In line with this hypothesis, we had previously demonstrate that bLf, like the proton pump inhibitors ConcA and BafA1, inhibits V-ATPase proton pumping and hydrolytic activities in crude membrane fractions and isolated lysosomes (21). Altogether, these data support the notion that the selectivity of bLf relies not only in the intracellular acidification caused by inhibition of plasmalemmal V-ATPase but also in the lysosomal V-ATPase dysfunction, which in this way amplifies its cytotoxic effects (Figure 7). These mechanistic insights further reinforce the bLf great advantage over other conventional V-ATPase inhibitors used as anticancer agents, namely the absence of secondary effects.

In another highly metastatic breast cancer cell line (4T1), known to exhibit V-ATPase at the plasma membrane, the combined treatment of Lf and tamoxifen suppresses the dissemination of lung and liver tumor metastases (39). Also, Lf administration significantly inhibits liver and lung metastasis produced by L5178Y-ML25 lymphoma cells and B16-BL6 melanoma cells, respectively (40). The recognized high levels of V-ATPase in cancer cells (32, 33) likely explains the anti-metastatic role of Lf against these other types of highly metastatic cancer cells. However, it would be interesting in the future to assess whether Lf induces lysosomal dysfunction in L5178Y-ML25 lymphoma and B16-BL6 melanoma cells, which also expose V-ATPase at the plasma membrane.

A recent study asserts that the abnormalities of extracellular acidification along with intracellular alkalinization of all types of solid tumors and leukemic cells appear to be a specific hallmark of malignancy (41). Attempts to induce intracellular acidification using proton transport inhibitors and other intracellular acidifiers, is thus becoming a new therapeutic strategy in cancer treatment. Notably, our finding that bLf acts as a specific inhibitor of V-ATPase at the plasma membrane of different highly metastatic cancer cells, while exerting no effect on non-tumorigenic cells is aligned with this novel concept. Besides, being bLf a natural compound means that its clinical usage surpasses multidrug resistance, which is a severe problem of current cancer therapies. Moreover, the dose used in this study is in the range used in several *in vivo* studies (42), therefore it is clinically relevant and it can be used for further analysis in *in vivo* models. Finally, as bLf is a commercially available non-toxic and low-cost dietary natural protein, the finding of V-ATPase as a selective and common molecular target of highly metastatic cancers brings novel important data for further *in vitro* and *in vivo* researches on the anticancer activity of bLf, and will ultimately contribute to its safer and more rational application as a nutraceutical in the human therapy of highly metastatic cancers.

## Author Contributions

Conceived and designed the experiments, analyzed the data, and wrote the papers: JG, CP, LR, and MC-R. Performed the experiments: JG and CP.: Contributed reagents/materials/analysis tools: LR and MC-R.

## Conflict of Interest Statement

The authors declare that the research was conducted in the absence of any commercial or financial relationships that could be construed as a potential conflict of interest.

## Abbreviations

BafA1, bafilomycin A1; BCECF-AM, 2′,7′-bis-(2-carboxyethyl)-5-(and-6)-carboxyfluorescein-acetoxymethyl ester; bLf, bovine lactoferrin; CF, carboxyfluorescein; CFSE, carboxyfluorescein diacetate succinimidyl ester; ConcA, concanamycin A; ECAR, extracellular acidification rate; Lf, lactoferrin; pHi, intracellular pH; PS, phosphatidylserine.
