# Supplementary material for: Bovine Milk Lactoferrin Selectively Kills Highly Metastatic Prostate Cancer PC-3 and Osteosarcoma MG-63 Cells In Vitro
Source: Front Oncol. 2018 Jun 4;8:200. doi: 10.3389/fonc.2018.00200 (PMC5994723; doi:10.3389/fonc.2018.00200)
Supplement: Supplementary file 1 [file Table_1.DOCX]

Supplementary Material

### Bovine milk lactoferrin selectively kills highly metastatic prostate cancer PC-3 and osteosarcoma MG-63 cells *in vitro*

Joana P. Guedes^1,2^, Cátia S. Pereira^1,2^, Manuela Côrte-Real^1*^, Lígia R. Rodrigues ^2^

^1^ Center of Molecular and Environmental Biology (CBMA), Department of Biology, University of Minho, Braga, Portugal

^2^ Center of Biological Engineering (CEB), Department of Biological Engineering, University of Minho, Braga, Portugal

*** Correspondence:**Manuela Côrte-Real, mcortereal@bio.uminho.pt

# Supplementary Figures and Tables

## Supplementary Table

| **Cell line** | **Cell Proliferation Inhibition (%)** | | **SD** |
| --- | --- | --- | --- |
| **MDA-MB-231** | Cisplatin | 11.56^§^ | 3.67 |
|  | bLf | 34.30^*§^ | 3.40 |
| **PC-3** | Etoposide | 30.45^§^ | 23.29 |
|  | bLf | 19.79^*§^ | 10.63 |
| **MG-63** | Cisplatin | 20.15^§^ | 14.15 |
|  | bLf | 34.00^*§^ | 13.03 |

**Table S1. Cell proliferation of the three highly metastatic cancer cell lines MDA-MB-231, PC-3 and MG-63 is similarly inhibited by bLf.** Inhibition of cell proliferation by bLf, cisplatin or etoposide follows an exponential kinetics. The percentage of inhibition (%) was estimated through the mean of the slopes of the linearised exponential functions of the CF mean fluorescence intensity of cells treated with 175 µM of bLf, cisplatin or etoposide (used as positive control for MDA-MB-231/MG-63 and PC-3 cells, respectively) *versus* time, normalized to the mean of the slopes of the linearised exponential functions of the CF mean fluorescence intensity of untreated cells (negative control), for each cell line. The values presented are the relative means expressed in percentage ± SD of three independent experiments: *^$^* sensitivity of each cell line to bLf in comparison with cisplatin/etoposide; * comparison of bLf sensitivity between the 3 cell lines. No significant differences were observed between the different conditions tested.
